# The Role of Sulfur in Agronomic Biofortification with Essential Micronutrients

**DOI:** 10.3390/plants11151979

**Published:** 2022-07-29

**Authors:** Styliani N. Chorianopoulou, Dimitris L. Bouranis

**Affiliations:** Plant Physiology Laboratory, Crop Science Department, Agricultural University of Athens, 11855 Athens, Greece

**Keywords:** copper homeostasis, iron homeostasis, manganese homeostasis, sulfur homeostasis, zinc homeostasis

## Abstract

Sulfur (S) is an essential macronutrient for plants, being necessary for their growth and metabolism and exhibiting diverse roles throughout their life cycles. Inside the plant body, S is present either in one of its inorganic forms or incorporated in an organic compound. Moreover, organic S compounds may contain S in its reduced or oxidized form. Among others, S plays roles in maintaining the homeostasis of essential micronutrients, e.g., iron (Fe), copper (Cu), zinc (Zn), and manganese (Mn). One of the most well-known connections is homeostasis between S and Fe, mainly in terms of the role of S in uptake, transportation, and distribution of Fe, as well as the functional interactions of S with Fe in the Fe-S clusters. This review reports the available information describing the connections between the homeostasis of S and Fe, Cu, Zn, and Mn in plants. The roles of S- or sulfur-derived organic ligands in metal uptake and translocation within the plant are highlighted. Moreover, the roles of these micronutrients in S homeostasis are also discussed.

## 1. Introduction

Iron (Fe), copper (Cu), zinc (Zn), and manganese (Mn) are essential micronutrients (EM) for plants, animals, and humans. Both their deficiency and excess cause various malfunctions in each organism. These elements comprise a metalome of central contribution to plant functioning. For humans to consume food of quality, the soil, plant, and human chain requires very efficient management of the ΕΜ metalome, through acquisition, transport, translocation, utilization, and re-translocation, by cofunctioning of the biological systems that will handle the EM within the plants. Understanding of the EM metalome homeostasis requires a detailed knowledge of the dynamics of this network in plants [1,2].

Soils can present multiple nutrient deficiencies in all classes of nutrients. The phytoavailability of soil EM to crops is influenced by various soil factors. Poor EM phytoavailability in soils causes a reduction in crop production and lower nutritional value of the crops’ products. Increasing the phytoavailability of EM to crops, and the EM content of crops during plant growth, is a process known as biofortification, and it is distinct to agronomic and genetic biofortification. Agronomic biofortification is the biofortification (or phytofortification) process that is based on the application of EM-containing mineral fertilizer to the soil and/or plant leaves (i.e., foliarly) toward increasing the EM contents of the edible part of the food crop. On the other hand, genetic biofortification is described as the biofortification process that involves classical breeding (or genetic engineering) to achieve this target [3,4,5,6,7,8,9,10,11,12,13].

Cakmak and Kutman [14] mention that “for foliar Zn applications to wheat the options are zinc sulfate and EDTA-chelated Zn. Zinc sulfate is at least as effective as Zn-EDTA for correcting Zn deficiency and increasing Zn concentrations in tissues, which means that it is the most cost-effective option compared with the relatively highly priced Zn-EDTA”. Specifically, zinc sulfate is mentioned, and not zinc nitrate, which raises the question of why sulfate is the accompanying anion.

Sulfur (S) plays crucial roles [15], including in the management of the ΕΜ metalome [16,17]. Each of the EM at first exists as a free cation, which is an existence that causes undesirable actions. Efficient handling of the EM metalome requires efficient chelation, transport, and translocation, along with efficient management of these actions. For each one of these management systems toward handling each EM properly, the phytoavailability of EM, along with the proper form of S in place and in time, is of central interest. Moreover, the functional EM metalome of plants is modified by S availability.

This review elaborates on the interactions between S and the micronutrients Fe, Cu, Zn, and Mn, and focuses on the levels of the cellular compartments, the various plant tissues, as well as the whole plant, toward highlighting their contributions to agronomic phytofortification.

## 2. Sulfur Homeostasis in Plants

S is a macronutrient essential for plant growth and development. It is required for the biosynthesis of cysteine (Cys), methionine (Met), and glutathione (GSH) and for several secondary metabolites, such as glucosinolates, as well as for the biosynthesis of proteins, cofactors, and vitamins. S-containing metabolites have central roles in the responses of plants to various environmental conditions [18,19,20].

S is taken up from the rhizosphere in the form of sulfate, via sulfate transporters localized in the cytoplasmic membranes of rhizodermal and outer cortex cells. Subsequently, sulfate will either be transferred to the root stele and loaded in the xylem to be transported to the shoot or it will be translocated into root plastids. Upon arrival of the sulfate to the aerial plant parts, it will be finally transferred into chloroplasts. At any time, depending on the needs of the plant, sulfate may be also transported into the vacuoles of the cells either of the roots or the shoot of the plant. All these transfers are made mainly through specific sulfate transporters, localized either in a cytoplasmic membrane or in a subcellular organelle’s membrane [19,21,22].

After entering in a root or shoot plastid, sulfate is assimilated into adenosine-5′-phosphosulfate (APS), which is then reduced into sulfite, and then sulfide, leading to cysteine biosynthesis. Cysteine is the key metabolite for subsequent biosynthesis of the S-containing organic compounds in plants, while a major pool of sulfur is GSH, a Cys-containing tripeptide. In parallel, APS will be phosphorylated to 3′-phosphoadenosine-5′-phosphosulfate, an intermediate metabolite used for sulfation reactions. Moreover, sulfite may also be used for sulfolipids biosynthesis into the plastid, or it may be transported to peroxisome, where it can be reoxidized to sulfate through the activity of sulfite oxidase [18,19,23,24].

The regulation of S homeostasis takes place predominantly during sulfate uptake and APS reduction, through transcriptional regulation of the sulfate transporters as well as of APS reductase (APR) isoforms. Several metabolites have been appointed to have roles in the control of sulfate assimilation: GSH serves as a negative regulator through feedback inhibition, while OAS is a positive regulator of the sulfate uptake and assimilation pathway [18,22,25]. Phytohormones also play outstanding roles in the regulation of S homeostasis, such as cytokinins as well as the “stress-related” hormones abscisic acid, jasmonic acid, and salicylic acid [18]. Furthermore, the roles of regulatory components of sulfate uptake and assimilation such as the transcription factor SLIM1, the sulfur-responsive element SURE, and the miR395 have been described [19]. Recently, an epigenetic regulatory mechanism involving the nuclear-localized MORE SULFUR ACCUMULATION1 (MSA1) methyltransferase has been identified [26].

## 3. The Biological Importance of Essential Micronutrients

EMs play central roles in the metabolism, growth and production, maintenance, abiotic and biotic stress tolerance, and as structural and functional components of metalloproteins if they are at an optimal concentration. EMs are transition metals, with Fe and Cu to undergo redox changes under biological conditions. At supraoptimal levels, they are toxic because they can cause oxidative stress due to the production of reactive oxygen species via Fenton reaction. To sustain the appropriate ion homeostasis, plants maintain equilibrium in EM homeostasis by the establishment and maintenance of stable linkages with appropriate organic ligands in a specific geometry [27,28,29,30,31]. The following structural and functional roles highlight the biological importance of each one of the EMs.

*Importance of Fe*—Fe is required in several cellular processes, including photosynthesis, respiration, and sulfur assimilation. Fe exists as Fe(III) or Fe(II) under physiological conditions, participating in electron transfer reactions of the cells. The bulk of Fe in plant cells is needed in mitochondria and chloroplasts, where the major sinks of Fe are Fe-S clusters and heme are, and it is stored in ferritin or in vacuoles.

Several proteins contain Fe as a cofactor, mainly in the form of Fe-S clusters. Such proteins belong to the electron transport chains of chloroplasts and mitochondria. In the chloroplasts, the major Fe-S proteins are photosystem I (PSI), cytochrome b6f complex, ferredoxin, nitrite reductase, sulfite reductase, and adenosine 5′-phosphosulfate reductase (APR). In mitochondria, the main Fe-S proteins are the complexes I, II, and III of the respiratory chain and aconitase in the citric acid cycle. The biosynthesis of these clusters requires tightly regulated provision of chelated Fe and reduced S, in the form of cysteine [31,32,33,34,35].

*Importance of Cu*—Cu is a redox-active transition metal and under physiological conditions in vivo, it exists as Cu(II) or Cu(I) and participates in many physiological processes. Cu ions act as cofactors in a variety of enzymes such as cytochrome c oxidase, Cu/Zn-superoxide dismutase (Cu/Zn SOD), ascorbate oxidase, plastocyanin, laccase, amino oxidase, and polyphenol oxidase. At the cellular level, Cu possesses key roles in photosynthetic and respiratory electron transport chains, C and N metabolisms, biogenesis of molybdenum cofactor, Fe mobilization, protection against oxidative stress, oxidative phosphorylation, transcription protein trafficking machinery, ethylene sensing, and cell wall metabolism [29,36,37].

*Importance of Zn*—Zn forms tetrahedral complexes with cysteine residues of polypeptide chains. Known roles include its presence in enzymes involved in protein synthesis and energy production. Zn is required for the maintenance and the structural integrity of membranes via its binding to membrane phospholipid and sulfhydryl groups, resulting in the protection of membrane lipids and proteins against oxidative damage. It is involved in signal transduction pathways via mitogen-activated protein kinases, and it plays an important role in seed development [32,38,39].

*Importance of Mn*—Mn presents the oxidation states II, III, or IV, and serves as a cofactor in various enzymes within a plant cell, where it can fulfill two roles in proteins: (1) as a catalytically active metal or (2) as an enzyme activator. Mn contributes to photosynthesis, defense against oxidative stress, lipid biosynthesis, nitrogen metabolism, gibberellic acid biosynthesis, and RNA polymerase activation. Representatives for the catalytic role are the Mn-containing water splitting system of photosystem II, the Mn-containing superoxide dismutase, and the oxalate oxidase. The group of the Mn-activated enzymes consists of PEP carboxykinase, isocitrate dehydrogenase, phenylalanine ammonia lyase, and malic enzyme. Proteins of this group are known to be involved in the shikimic acid pathway, as well as the biosynthetic pathways of aromatic amino acids, flavonoids, lignins, and the indole acetic acid. Τhe role of Mn in the activation process is less specific and in many cases it can be replaced by magnesium [32,40,41].

## 4. Functional Interactions between EM and S

**Figure 1:**
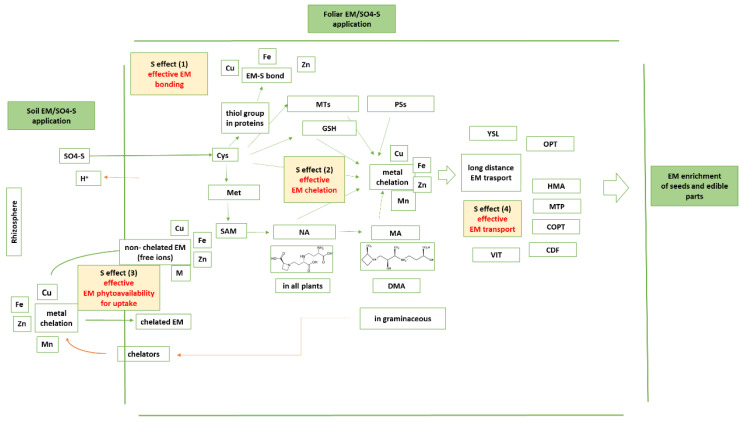
The contribution of S homeostasis in essential micronutrients’ homeostasis within plants toward efficient seed EM loading.

*The EM-S bonds*—The first level of interaction between EM and S is the formation of an effective EM-S bond (Figure 1). Ligands are distinguished as weakly (or hard) vs. highly (or soft) polarizable ones. The weakly polarizable ligands include the carboxylate groups with negatively charged oxygen atoms and the carbonyl groups with polar oxygen atoms. The highly polarizable ligands include the sulfhydryl groups. Aspartate (Asp) and glutamate (Glu) participate with their carboxylate groups; asparagine (Asn) and glutamine (Gln) participate with their carbonyl groups; while Cys and Met, the S-containing amino acids, are soft ligands. Histidine (His) carries the imidazole ring that contains the borderline aromatic N.

*The contribution of S in the bond*—The S atom is a soft donor; hence, it prefers to bind with soft cations. In this way, it provides high redox potential to metal redox couples. The reduced form of the metal cation is softer than the oxidized one. Between two metal centers, the S-containing ligands can function as bridging ligands (M-S-M) or as monodentate ones (M=S). In proteins, S is found as thiolate or thioether. Thioethers are organic sulfides (C-S-C). Examples of sulfides are Met and biotin. Thiolates contain the thiol group (C-S-H).

*The Fe-S bond*—Ligands are electron donors and the metals’ electron acceptors. The softer Fe(II) catalyzes the Fenton reaction with hydrogen peroxide; hence, an efficient bonding is needed. Fe-S proteins are characterized by the presence of Fe-S clusters, which are found in a variety of metalloproteins. These clusters contain di-, tri-, and tetra-Fe centers and are sulfide-linked in variable oxidation states. Fe-S clusters contribute to the oxidation-reduction reactions of electron transport chains. There are several proteins containing Fe-S clusters that regulate gene expression. In most Fe-S proteins, the terminal ligands are thiolate sulfur centers from cysteinyl residues. The Fe centers are tetrahedral, while the sulfide groups are two- or three-coordinated [42].

*The Cu-S bond*—The harder Cu(II) can be co-ordinated by oxygen and nitrogen atoms of the harder amino acids (Tyr, Thr, His). The Cu(II)-N bonds are stable and often inert, while the Cu(II)-O bonds are more labile. The soft Cu(I) is stabilized by soft ligands. Cu(I) catalyzes the Fenton reaction with hydrogen peroxide. Cu(Ι) in proteins prefers the S atoms or ions of Cys and Met. Cu-thiolate and Cu-thioether bonds are found in a wide variety of enzymes with multifaceted co-ordination chemistry. S ligation to the metal centers provides special properties to Cu enzymes. S atoms from thiolates or thioethers act as donor ligands in a variety of Cu complexes [43].

*The Zn-S bond*—Zn is classified as borderline metal; i.e., Zn(ΙΙ) does not act as either hard or soft. Moreover, Zn does not have a strong preference for O-, N-, or S-coordination. In the various plant biological systems, Zn exists only as Zn(II), not taking part in redox reactions. In protein Zn-binding sites, the Zn(II) may be co-ordinated by the oxygen of aspartate or glutamate, the nitrogen of histidine, or the sulfur of cysteine. Among these, His is the most observed, followed by Cys, through which S plays a functional (catalytic) and a structural role in enzyme reactions. The Zn-S bond serves several roles: in metallothionein and Zn release, in thionein and Zn binding, in control of Zn transfer reactions and availability of cellular Zn in mononuclear sites, in the cellular distribution of Zn, in proteins that detect the availability of cellular Zn, in redox-active Zn proteins, in Zn thiolate cluster structures, and in Zn co-ordination dynamics [44].

*The Mn-S bond*—There are no Mn metalloenzymes containing an Mn-S co-ordination sphere, as Mn(II) establishes co-ordination with hard ligands. His is the important ligand for Mn(ΙΙ), while Cyst and Met are less likely to co-ordinate with Mn(ΙΙ) [40].

## 5. The Chelation Process

### 5.1. The Need for Chelation

Free EM can be toxic; therefore, EMs in plants exist as free ions in either very small amounts or not at all. Instead, the metals that are present in plant fluids must be in less-reactive chemical forms, bound to “proper” organic compounds, to prevent the uncontrolled binding. Selected organic molecules are implicated in metal ion binding and are known as metal ion ligands or chelators. Chelation improves acquisition and transport of EM with low solubility, along with immobilization, toward EM storage and tolerance. The formation of EM complexes provides solubility as well as protection during long-distance transport, as the EM atom is surrounded by the ligands. These chemical species donate a number of electron pairs to the EM to form the complexes.

### 5.2. Chelators

Possible candidates as ligands are several small molecules: Organic acids offer their carboxylate groups for chelation, among them citrate and malate. The amino acids histidine (His), cysteine (Cys), nicotianamine (NA), and the phytosiderophores (PSs), the high-affinity Fe(III) chelating compounds derived from NA, i.e., mugineic (MA) and 2′-deoxymugineic (DMA) acids, count as well. Peptides and proteins (e.g., metallothioneins) are also included.

### 5.3. Sulfur-Based Chelators

Sulfur interacts with EM at the chelation level [45], and in this section, the corresponding chelators are summarized. Therefore, the second level of S and EM interactions is the formation and action of S-based chelators (Figure 1). These chelators may contain and utilize the sulhydryl group or may give genesis to chelators containing, and contributing with, the carboxyl group. Some of the chelators are excreted into the rhizosphere toward effective EM phytoavailability, which comprises a third level of S and EM interactions.

#### 5.3.1. Cysteine

Cys contains thiol and it can act directly as a metal-chelator. It combines EM binding properties along with catalytic activity and redox properties. These properties of the thiol group are interdependent and permit the redox regulation of proteins, metal binding, and the control of redox activity by the respective metal, as well as the control of metal-based enzyme catalysis by the ligands. In proteins, cysteine contributes also to “redox switches” and to sensing concentrations of oxidative stressors, taking part in key regulatory and signaling pathways. Additionally, it participates in the biosynthesis of phytochelatins [46].

#### 5.3.2. Nicotianamine

NA has been shown to participate in the transport of the EM Fe, Cu, Mn, and Zn. NA is produced by methionine and acts as a chelator through its carboxyl groups, possessing a special role in the interaction between S and EM homeostasis. NA is synthesized by NA synthase (NAS) from S-adenosyl-L-methionine. It is a ubiquitous metal chelator in all plants. NA is an Fe chelator, and it has been demonstrated its ability to bind both Fe and Cu. It is believed to play a primary role in EM homeostasis, and therefore it links EM homeostasis to S homeostasis. In vitro, NA can form stable complexes with Fe, Cu, Zn, and Mn. The maximum stability of all EM-NA complexes is observed at pH 6.5. This renders NA more likely as a symplastic chelator of metals. Cu is the exception because Cu-NA complex is stable in mild acidic conditions. This fact favors the probable presence of Cu-NA complex in the xylem [47,48,49,50].

Under Cu, Fe or Zn deficiency, NA synthase (NAS) genes are upregulated in roots and shoots of plants. The essentiality of NA has been established for the EM transport in veins and interveinal areas, also for reproductive growth and fertility. These suggest a role for NA in long-distance translocation of EM. The overexpression of a Hordeum NAS in transgenic tobacco plants resulted in an elevated Cu, Fe and Zn content in leaves and flowers and higher Fe and Zn content in pollen and seeds [51]. The Cu-NA, Fe-NA, and Zn-NA complexes in the phloem, and the Cu-NA and Zn-NA ones in the xylem support the translocation of EM from roots to shoots [46,47,49].

#### 5.3.3. Mugineic Acid and Its Derivatives

MA has been shown to participate in the transport of the EM Fe, Cu, Mn, and Zn. MA consists of an azetidine group and three carboxylates. It is a phytosiderophore (PS), i.e., a plant-produced siderophore, functioning as an Fe accumulating agent. Τhe precursor is NA, and the ability to modify NA into MA and derivative compounds is unique to graminaceous species [2]. Those derivative compounds carry modifications on their molecules (mainly hydroxylations) toward and increasing stability in low-pH environments [52]. The family of PSs consists of eight compounds [53]. PSs are excreted by graminaceous plants into the plant’s rhizosphere, they chelate Fe(III), and then the Fe(III)-PS complex is transported into the root (Strategy II for Fe uptake).

The nongraminaceous species do not synthesize PSs for iron uptake. These plants solubilize soil Fe(III) by secretion of organic acids and H^+^ to the rhizosphere. This Fe(III) is then reduced by ferric-chelate reductases (FRO) localized in the plasma membrane, and the resulting Fe(II) is transferred into root cells (Strategy (I) for Fe uptake).

There are, however, some plant species known to be exceptions to the Strategy (I)/(II) classification, and instead use a combined method. The Strategy (I) Fe(II) transporters OsIRT1 and OsIRT2 have also been discovered in rice. Moreover, Tsednee et al. [54] described that *Arabidopsis halleri* utilizes NA as an excreted Fe-chelating agent, resembling the Strategy (II) secretion of PSs. Finally, the olive tree seems to have Strategy (II) method for Fe uptake, although it is a nongraminaceous plant species [33,55].

#### 5.3.4. Glutathione

The aforementioned molecules are small ones. Moving to peptides, GSH is the tripeptide γ-Glu-Cys-Gly, a thiol compound with major interest as an antioxidant molecule. GSH is widely distributed in plant cell compartments, in a range of 0.5–10 mM. It performs key roles in the antioxidant machinery and cellular redox homeostasis, as well as in the defense mechanism of plants. GSH is involved in the chelation and detoxification of free EM, and the maintenance of a high GSH level is required to perform these roles, among which is the reduction of oxidative damage caused by accumulated metals [56,57,58,59,60,61,62,63,64,65,66,67,68].

#### 5.3.5. Phytochelatins

Phytochelatins (PCs) are derived from glutathione (γGlu-Cys)n-Gly, produced by the enzyme phytochelatin synthase, where GSH is a precursor for their synthesis. PCs act as chelators, which are important for the detoxification of heavy metals. Depending on the number of the γGlu-Cys group they carry, they are abbreviated as PC2 through PC11. Apart from Gly (PC), the third component may be Ala (homo-PC), Ser (hydroxymethyl-PC), Glu or Gln (iso-PC), or none (desglycine-PC). Synthesis of PCs may be metal-specific and/or plant-specific. Fe has been reported as a strong inducer of PCs, while Zn is a weak inducer and Cu is a moderate one [69,70]. The EM ions, Zn(II), Cu(II), and Mn(II) may be transferred into the vacuoles as forms of PC2-EM complexes [71,72].

#### 5.3.6. Metallothioneins

Metallothioneins (MTs) are polypeptides of low molecular weight (4–14 kDa), rich in Cys. MTs are a class of metal chelator thiol compounds and are S-donor ligands. MTs bind EM through the thiol group of their Cys-residues. MTs are products of mRNA translation, and their expression is regulated during metal stress toward EM detoxification and maintaining their homeostasis [73,74,75,76,77,78].

The ability of MTs to bind and sequester with EM depends upon the distribution of Cys residues. It has been proposed that the MT is composed of the α and β binding domains, each one composed of various Cys-clusters. The α-domain is the C-terminal part that can bind four divalent metal ions. The β-domain is the N-terminal part of the peptide, which has three binding sites for divalent ions. The two domains are Cys-rich, separated by a central Cys-free spacer. The Cys residues are arranged in various motifs, such as Cys-Cys, Cys-X-Cys, and Cys-X-X-Cys sequences, where X represents another amino acid. Abiotic stresses, such as heat, drought, light, salinity, and senescence, can modify MT gene expression. The MT family includes a number of small Cys-rich proteins holding the ability to co-ordinate transition metal ions, such us Zn(II) and Cu(I) [8,73,78,79,80,81].

MTs show high affinity for EM through the involvement of peptide donor groups (S-thiol and N-imidazole) in metal chelation. Metal atoms are covalently bonded via sulfhydryl residues of cysteines and MTs provide thiols in their reduced state for metal chelation. Zn and Cu strongly induce MT gene expression in plants. The high affinity of MTs for metals provides a protective mechanism against metal toxicity, especially against Zn and Cu toxicity, significant for the maintenance of their homeostasis [78,79,82,83,84,85].

## 6. Transporters of Metal Micronutrients

Sulfur interacts with EM at the translocation and transport levels, too, and in this section, the corresponding transporters for handling of the EM co-ordinated by the S-based chelators are summarized. This comprises the fourth level of S and EM interactions (Figure 1).

A number of gene transporter families are involved in metal micronutrients’ transport in plants. These transporter families include: Yellow Stripe-Like (YSL), Zinc regulated transporter/Iron-regulated transporter [ZRT/IRT1]-related Protein (ZIP), Natural Resistance Associated Macrophage Protein) (NRAMP), Heavy Metal ATPase (HMA), Metal Tolerance/Transport Protein (MTP), Copper Transporter (COPT), and Vacuolar Iron Transporter (VIT).

### 6.1. Yellow Stripe-like

The YSL transporters belong to the OligoPeptide Transporter superfamily (OPT). The members of the OPT superfamily transport tri-, tetra-, penta-, and hexapeptides. YS and YSL proteins mediate the uptake of metals complexed with the plant-derived metal-ligands known as NA or PSs [49]. Τhe most notable representative of this family is ZmYS1, the protein of maize known to transport mainly Fe-PS from the rhizosphere inside plant roots. ZmYS1 is overexpressed under Fe-deficient conditions, but plays a role in the uptake of Cu, Zn, and Mn complexes with mugineic acid [86,87,88,89].

YSL proteins function mainly as transporters of EM-NA complexes inside the plant body, having roles in the translocation of those complexes inside the vasculature toward the developing tissues, as well as in the metal loading of the developing seeds. These roles suggest multiple functions of YSL proteins in Fe, Cu, Zn, and Mn long-distance transport through vascular tissues to the whole plant body. It has been suggested that YSL transport divalent cations are complexed with NA in the phloem [49]. Rice OsYSL2 has been implicated in the transport of Fe(II)-NA and Mn(II)-NA complexes but not with Fe(III)-NA [90]. Another member of the rice YSL family, OsYSL6 is required for Mn transport and sequestration from the apoplast into the symplast, especially when toxic levels of this metal are present in plant rhizosphere [91].

YSL proteins also have the nongrass plants, which seem to not produce PS for Fe uptake and transport. In these plants, YSL probably functions as metal-NA transporters. *Arabidopsis thaliana* AtYSL1 is overexpressed when plants are grown under high Fe conditions, as well as during leaf senescence [92]. AtYSL2, on the other hand, is expressed under Fe, Cu, or Zn sufficiency conditions [93,94].

### 6.2. Zinc Regulated Transporter/Iron-Regulated Transporter [ZRT/IRT1]-Related Protein

The ZIP family is found in all eukaryotes [95]. ZIP transporters are involved in the uptake, intracellular transport, and detoxification of various divalent cations, including Zn^2+^, Fe^2+^, Cu^2+^, and Mn^2+^, in plants. ZIP proteins assist metal ion homeostasis by mediating the transportation of those cations into the cytoplasm. ZIP transporters are located in the membranes of various cell organelles, and they are known to play a pivotal role in Zn homeostasis of the plants. Members of the ZIP family are the Fe-regulated transporters (IRTs), which are the major Fe transporters in nongrass plants. The genes of the IRT family exhibit increased expression levels under Fe deficiency conditions, and they are expressed under different metal stresses [28,96,97,98].

*Arabidopsis thaliana* AtIRT1 is the main Fe(II) transporter for Fe uptake from the rhizosphere [99,100,101,102], while AtIRT2 also seems to function for the uptake and transport of Fe(II) [103]. IRTs of Arabidopsis are implicated in the transport of divalent metal ions such as Zn(II) and Mn(II). AtIRT3 is involved in Fe and Zn translocation, and overexpression of AtIRT3 resulted in increased accumulation of Fe in roots and Zn in shoots [104]. Among *A. thaliana* ZIP transporters, AtZIP1-AtZIP5, AtZIP9-AtZIP12, and AtIRT3 seem to function in Zn acquisition under Zn-deficient conditions [105].

Grasses also have functional ZIP transporters. Rice OsIRT1 is a functional Fe transporter for the uptake of Fe from the rhizosphere, contributing also to Fe translocation to the shoot and seeds [106]. As opposed to this, the accumulation of OsZIP4 and OsZIP5 results in increased Zn concentration in roots but not increased Zn content in seeds [107,108]. Barley IRT1 is also able to transport Mn and Fe, but it seems that its primary function here is Mn acquisition from the soil [109].

### 6.3. Natural Resistance Associated Macrophage Protein

The NRAMPs are metal transporters, and they are found in all living organisms. They are known to be involved in divalent metal uptake into the cell and subsequent intracellular transportation into the various organelles [110]. The members of the plant NRAMP family contribute to the homeostasis of Fe(II), Mn(II), Cu(II), and Zn(II) inside the cell. NRAMP transporters also translocate toxic metals such as arsenic (As^3+^), lead (Pb^2+^), and cadmium (Cd^2+^) [111,112]. *Arabidopsis thaliana* AtNRAMP1, AtNRAMP3, and AtNRAMP4 play crucial roles in the transportation of Fe(II) and Mn(II) [113,114]. Rice OsNRAMP3, OsNRAMP4, and OsNRAMP5 have roles in the transport of Mn(II): OsNRAMP3 is considered responsible for Mn allocation from source to sink tissues, while OsNRAMP4 and OsNRAMP5 have functions in intracellular mobilization of Mn [115,116,117]. Under limiting Mn supply, OsNRAMP3 switches the flow of Mn toward the young leaves and panicles. When the Mn supply is at toxic levels, the NRAMP3 protein is degraded, and Mn is directed to older leaves.

### 6.4. Heavy Metal ATPase

The HMAs (or P1B-type ATPases) play crucial roles in Zn and Cd translocation or detoxification in plants. They are a diverse group of proteins, both in terms of tissue distribution and subcellular localization, as well as in terms of metal specificity. HMAs are divided into two subgroups based on their metal-substrate specificity: a Cu/Ag subgroup and a Zn/Co/Cd/Pb subgroup [118,119]. *Arabidopsis thaliana* AtHMA1–AtHMA4 belong to the Zn/Co/Cd/Pb subgroup [120,121,122]. AtHMA1 is localized to the chloroplasts [123], while AtHMA3 is localized to the tonoplast for the efficient detoxification of Cd and Zn via vacuolar sequestration [124,125]. AtHMA2 and AtHMA4 are both localized to the plasma membrane of the cells adjacent to xylem vessels of roots and function in Zn and Cd efflux from cells [126,127,128,129]. AtHMA5-AtHMA8 belong to the Cu/Ag subgroup. AtHMA7 is considered to have a role in Cu delivery to ethylene receptors [130]. AtHMA6 has a function in the delivery of Cu to the plastid, especially to the Cu-dependent proteins plastocyanin and Cu/Zn SOD [131]. Rice OsHMA2 belongs to the Zn/Co/Cd/Pb subgroup, having an important role in long-distance transport of Zn and Cd, and participating in Zn and Cd translocation to developing seeds [132]. OsHMA3 belongs to the same subgroup and transports Cd, having a role in the transportation of Cd into vacuoles of root cells [132,133].

### 6.5. Metal Tolerance/Transport Protein

The MTP members belong to the Cation Diffusion Facilitators (CDF) superfamily. These transporters are believed to be responsible for various metal ions’ homeostasis, including Fe, Mn, and Zn. These proteins are commonly involved in the translocation of metals out of the cytosol into the organelles or toward the apoplast [134,135]. *Arabidopsis thaliana* AtMTP1 is a tonoplast-localized transporter which is expressed throughout the plant body, transporting Zn into vacuoles [136,137]. Moreover, AtMTP3 and AtMTP8 transport Zn and Mn, respectively, playing a pivotal role in metal tolerance [138,139,140,141]. AtMTP11 functions by transferring Mn into endosomal vesicles, having a role in Mn transport and tolerance [142,143], while AtMTP12 has been identified as a Zn transporter [144]. The tonoplast-localized OsMTP8.1 and OsMTP8.2 enhance Mn uptake and tolerance through the sequestration of the metal into the vacuoles of the shoot and the root [145,146], and OsMTP11 is a transporter responsible for Mn absorption and translocation in rice plants [147].

### 6.6. Copper Transporters (COPT)

The major group of proteins implicated in Cu transport is the COPT proteins, which are localized either in the plasma membrane or in tonoplast and lysosome membranes. COPT proteins are highly specific for transport of Cu(I) but not for Cu(II) [148,149]. Therefore, membrane metalloreductases need to catalyze the reduction of Cu(II) to Cu(I) before Cu(I) transport with COPT. After passing through the transporter pore, an efficient delivery of Cu(I) to membrane-associated Cu-chaperones is needed [148,149]. *Arabidopsis thaliana* AtCOPT1, AtCOPT2, and AtCOPT6 exhibit higher expression levels in leaves, AtCOPT3 and AtCOPT5 in stems, and AtCOPT4 in roots [150,151,152]. AtCOPT5 is a tonoplast-localized protein, which is probably involved in stored Cu redistribution [151].

### 6.7. Vacuolar Iron Transporters (VIT)

The two major Fe storage cellular sites in plants are the vacuoles and ferritin. Ferritin is a protein which localizes in plastids, where Fe is accumulated in a bioavailable form [153]. On the contrary, vacuolar Fe strongly binds with phytates, resulting in a hardly bioavailable Fe form. Nevertheless, vacuolar accumulation of Fe represents an important cellular Fe homeostasis mechanism in plants. Vacuolar Fe compartmentalization and sequestration is mediated by VIT. VIT play significant roles in Fe homeostasis, especially under high Fe conditions, where VIT can prevent toxicity at the cellular level by maintaining optimal concentrations [154,155,156]. AtVIT1 can transport excess Fe into vacuoles when Arabidopsis seedlings grow under high Fe conditions [157]. OsVIT1 and OsVIT2 are overexpressed in the flag leaf blades and sheaths of rice plants, for efficient handling of Fe transport into the vacuoles [158].

## 7. Interactions of EM Homeostasis

Maintaining the homeostasis of each EM within the plant is a complex and dynamic task at all levels. Optimal concentrations must be maintained for safe functioning within the cells and organelles, which implies that deficiency or excess must be avoided. Moreover, each EM interacts and influences the functions of the others. Hence, tight regulation is needed, because suffering from deficiency or excess of each EM implies impaired cellular metabolism. Reduced growth and development are anticipated, coupled with imbalance in the uptake of other EMs and hectic tolerance to diseases [31,159,160,161,162,163].

The membrane transport systems are among the first that take part in the regulation of crosstalk between EMs. In the rhizosphere, the divalent forms of EM compete for universal metal transporters such as IRTs or NRAMPs for uptake. Within the plant, EMs compete for transport, binding, and storage. IRT1 and IRT2 support the entries of Fe(II), Zn(II), and Mn(II), while NRAMPs support the entries of Fe(II) and Mn(II). The Fe-regulated transporters of the ZIP family OsZIP1–4, as well as the transporter OsHMA2 which belongs to the heavy metal ATPase family, may also transport Fe(II) or Zn(II) [28,31,164,165,166].

In this line, EM binding proteins tend to select EM ions in an order of preference. The competitive EM must be excluded from binding sites for the proper ions to get their place. Mismetallation is one reason for impaired metabolism. EM crosstalks is an open field for research [28,167].

### The Contribution of S to EM Metalome Homeostasis

S deficiency reduces plant growth, which in turn has negative effects on the root uptake of other nutrients, such as, for example, N, K, and Mg, and vice versa: N, K, or Mg deficiencies reduce the uptake of S. Legumes have high requirements for S, and interaction between N and S can be found at the nodules. During the synthesis of the Mo co-factor, an interaction exists between S, Cu, Fe, and Zn. Mo uptake has been shown to be strongly increased under S deficiency, and to a smaller extent under Fe, Zn, Mn, or Cu deficiency [17]. In particular, the mitochondria are important players in nutrient interplay in plants, and the EMs are among the most important members of the mitochondrial metalome.

S interacts with Fe, while Fe, Cu, and Zn strongly interact with each other [168,169,170]. Hence, below, we elaborate on these interactions.

Due to the role of S in Fe uptake and transport within the plant, Fe deprivation induces a rebalancing of S metabolism to cope with increased needs for more efficient Fe mining and distribution. In this line, the response of graminaceous plants growing under Fe limitation is associated with S limitation. A connection point of Fe and S homeostasis is the Fe-S clusters. APS-reductase and sulfite reductase, two of the enzymes of S-assimilation pathway, are metalloenzymes containing Fe-S clusters, and are examples that highlight the role of Fe in S assimilation.

Fe ligands have a wide range of affinities, and this trait affects Fe homeostasis. The regulation of EM homeostasis and the maintenance of a stable metabolism requires complex crosstalk pathways in cells (for review see [31]), and below, we summarize these crosstalks. In particular, Fe competes with the other EMs during uptake, transport, and chemical reaction. Fe and Zn interact due to the similarity between both their divalent cations as well as their transporter proteins. As regards to their uptake and distribution, an antagonistic relationship between them has been reported. Zn concentration influences Fe uptake. Higher concentrations of Cu in relation to Zn due to the application of a Cu fertilizer in the rhizospheric solution can reduce Zn phytoavailability, and vice versa. This is due to the competition for the same transporters for absorption by the root. On the other hand, the use of Zn fertilizers has been described to have an impact on Cu concentration in wheat tissues. Both Fe and Cu function as cofactors for components of the cells’ electron transport chains. Fe and Cu interact and influence the uptake of each other. Antagonism also exists between Fe and Mn. During transport, it is their ratio in the solution that is more important compared with their absolute amounts.

## 8. From Rhizosphere to Seed

After taken up from the rhizosphere, EM and S follow a complex path through many different membrane systems and plant compartments. For an EM, or an S, form to be utilized by the seed, the route includes availability in the soil for uptake by the roots (phytoavailability) and entry into roots; transport and translocation within the vasculature, including xylem loading in roots; xylem unloading and phloem loading in the leaves; and finally, phloem unloading in the developing organs and seeds [48,49,171,172,173,174,175,176].

Once an EM is taken up into roots, it continues apoplastically or it enters a symplast. Then, forwarding of the EM requires movement from this symplast to a new one. This process includes either movement of the EM through plasmodesmata, or it must leave the first symplast by entering the apoplast outside the cells, and then enter again in another symplast. This represents a retardation in the translocation of the EM within the plant. The membrane potential of the plasma membrane is negative inside, maintained by the plasma membrane H^+^-ATPases. Hence, the membrane potential is the major driving force for the passive uptake of positively charged EM ions into the cell. When inside the cytoplasm, the tonoplast has an inside positive membrane potential, and in this case, active transport systems are required for import and export from the vacuole [4,175,177].

In this route to the seed, we will highlight the contribution of sulfur and the S-based contributing components and mechanisms to the movement of an EM.

### 8.1. Bioavailability for Uptake

The rhizosphere is the source of sulfate and EM for plants; thus, efficient sulfate and EM uptake systems by plants are required. The phytoavailability of an EM depends upon its solubility in the rhizosphere, and this attribute varies considerably according to soil composition, pH, and available P [38].

Fe in aerated soils is poorly phytoavailable; it is present as Fe(III) and precipitates by forming poorly soluble oxides, hydroxides, and oxyhydroxides not readily available for plant uptake. The connection between the redox Fe switch and S has been reviewed by Li et al. [178]. To uptake Fe from the rhizosphere, plants have developed two different strategies. Based on this trait, plants are divided into two groups: graminaceous and nongraminaceous plants. Nongraminaceous plants have adopted the reductive strategy (the Strategy I) for Fe uptake from their rhizosphere. This strategy includes rhizosphere acidification, along with secretion of chelators, toward chelating Fe(III). Graminaceous plants have adopted the chelation strategy (the Strategy II) for Fe uptake. This strategy is based on the release of PSs, that act as Fe(III) chelating compounds synthesized from Met via NA. As such, this strategy is inevitably linked to S homeostasis. In this line, in a graminaceous plant facing S deficiency, the release of PSs is reduced [45].

It is still obscure how the Cu ions are actively mobilized by the plant. PS secretion by graminaceous plants enhances Cu mobilization, but so far, no evidence is available for the uptake of Cu-PS complexes by the root. Zn and Mn are taken up as the free divalent ions. Zn uptake is not closely related to the Zn concentration in the soil solution [38,179].

### 8.2. Entry into Roots

The root cortex is the first symplastic domain EM enters. When inside root cells, the EM binds to the existing organic molecules and travels bound to a symplastic EM chelator. The EM-NA complex is transportable; it diffuses between cells of the root symplast, through the plasmodesmal connections, and toward the xylem. The transportation of the EM in the root symplast is restricted by the storage into the vacuole, and this import requires active transporters [175].

*Fe uptake*—The components of the reductive strategy for Fe uptake include: H^+^-ATPases for root apoplast and rhizosphere acidification, transporters involved in Fe(III)-chelators secretion, Fe(III) chelate reductase for the reduction of Fe(III) to Fe(II), and Fe(II) transporters for the translocation of Fe(II) across the plasma membrane inside the root cells. The components of the chelation strategy are: enzymes involved in PSs (Fe(III) chelators) biosynthesis, PSs exporters to root apoplast and rhizosphere, and transporters for the translocation of Fe(III)-PS complexes inside the root cells. This division presents exceptions, the most known of which is the case of rice. Rice follows a combined strategy: apart from the components of the chelating strategy, it also utilizes Fe(II) transporters for the translocation of Fe(II), which is the predominant Fe species in rice fields. It is evident though that the two strategies are not as different as it was initially assumed. Both of them include the secretion of an Fe(III) chelator, which will chelate Fe(III). Then, they either facilitate its reduction to Fe(II) and translocation of Fe(II) inside the cells, or they mediate the translocation of the resulting complex directly inside root cells.

*Zn uptake*—Ιn nongraminaceous plants, Zn(II) uptake into root cells is mediated by IRT3, which also transports Fe(II) in addition to Zn(II). IRT1, the uptake transporter for Fe, also has a broad substrate range and uptakes Zn(II) too. Graminaceous plants release MA for Fe(III) uptake, and may employ this mechanism toward acquiring Zn. MA chelates Zn(II) in addition to Fe(III). The Fe(III)-MA transporter YS1 transports the Zn(II)-MA complex as well.

*Mn uptake*—Mn is taken up as Mn(II) into the root epidermal or cortical cells by various transporters, among which is the NRAMP [180].

### 8.3. Transport within the Vascular System

The EM species existing in any given compartment determines its biological activity. According to Alvarez-Fernandez et al. [45], the existing information on the actual EM complexes of the plant fluids is still hectic because the metal speciation affects most of the physicochemical and physiological traits, such as solubility, precipitation, acid-base equilibria, diffusivity, electron-transfer reactions, and the possibility for EM toxicity. These traits also affect the capability of the EM complex to be a substrate of membrane transporters for loading and unloading to xylem and phloem.

#### 8.3.1. Transport in the Xylem Vascular Tissue

The xylem contains slightly acidic sap with a pH level of around pH 5–5.5. To enter the xylem, EM is actively exported from the symplast. Entering the xylem sap, EM is transported complexed or as a free cation. In this sap, carboxylic acids exist at concentrations from 2 to 9 mM.

It is not known how the Fe complexes enter into the xylem sap, where the Fe-citrate complex is localized. NA is not essential for xylem Fe transport. In graminaceous plants, PSs could also serve as Fe chelators in the xylem. NA seems to be the player for xylem Fe unloading. The Fe(II)-NA complex mediates the long-distance Fe allocation between plant organs. It has also been suggested that Fe-NA may be unloaded into the xylem by a YSL transporter [173].

Zn exists either as free hydrated Zn(II) ions, or co-ordinated with citrate and/or malate. The Zn-NA complex is involved in long-distance transport. Histidine has been proposed as a Zn ligand within cells. Zn-phytate complexes have been found in roots, whereas Zn-malate and Zn-citrate are the major species in shoots.

Cu(II)-DMA, but not Cu(II)-NA, has been detected in xylem sap of rice plants, although NA and DMA were found in the xylem and in comparable concentrations [45,47,181].

An EM is not transferred directly to the developing seed through the xylem. It is first allocated to the leaves. Then, it is exported from the xylem into the parenchyma and mesophyll cells. The transport proteins in action are not fully characterized. In turn, the EM is transferred out of the mesophyll cells toward the leaf apoplast, and it is loaded into the phloem, which is the vascular pathway to the developing seeds. In the graminaceous species, vascular bundles of the stem favor a more direct transfer of EM from the xylem to phloem strands for the transportation to the panicle [182].

#### 8.3.2. Transport in the Phloem Vascular Tissue

Loading of EM into the phloem occurs in leaves. The distribution of EM to developing organs depends on phloem transport, while the phloem is the only route for an EM to enter the developing seeds. When demand starts to increase, the EM is remobilized from senescing leaves toward allocating to the reproductive tissues. During EM remobilization, required active transport is not required for EM to exit the vacuole because membrane potential inside the tonoplast is positive.

The phloem sap is alkaline, with pH values ranging between 7 and 8, where EMs are sparingly soluble. Due to the alkaline pH of the phloem, an EM travels in the phloem bound to a chelator, most likely the NA. EM complexation with suitable chelators provides solubility and shielding for efficient phloem transport of EM to their sinks. Fe and Cu are highly reactive species, and they can easily undergo changes of valence, thus favoring the production of ROS [49,183].

Fe-containing compounds or complexes, and the Fe(III)-DMA complex, were detected in the phloem sap. The Fe(II)-NA complex has not yet been found in the phloem sap. It seems that NA is significant in Fe loading to the phloem, and once transferred into phloem, Fe may be transported in another chelated form, such as bound to proteins [45,184].

The phloem sap of rice contains the complexes Cu(II)-NA, Cu(II)-His, and high molecular-weight compounds. The Cu-containing proteins detected in phloem sap so far include a Cu-chaperone (CCH homolog), Cu/Zn-superoxide dismutase, and several MTs [180,185,186].

During the period of grain loading, the remobilization of Zn in the graminaceous species is not restricted to leaves; it also occurs from the stems, peduncles, florets, and rachis. How Zn is transported out of leaf mesophyll cells and how Zn enters the phloem is far from well-known. Almost all Zn in the phloem sap of rice was identified as the Zn(II)-NA complex [175,183,187].

Hectic information on the chemical forms of Mn in the phloem sap is available. In *R. communis*, Mn was detected in association with low molecular weight peptides [175].

*EM in the apoplastic fluid*—The apoplastic fluid plays important roles in the transport and storage of EM, and its composition is dependent on the import via xylem, the following absorption by cells, and finally the export by phloem. The available information is very hectic regarding tackling direct EM speciation on apoplastic fluid. For example, there are indications that Fe is present in the leaf apoplastic fluid as Fe-citrate complexes [45,188,189,190,191].

### 8.4. Unloading in Developing Seeds

*The EM content of the seed*—Several mechanisms are involved in phloem unloading and postphloem movement of EM in the developing seed. These include the movement through apoplastic barriers. The loading rates of EM imported through the phloem are regulated by translocation processes localized in both the sources, i.e., the leaves and the stems, and the seed sinks [192].

Phloem unloading in the developing seed seems to be symplastic into a specific domain, with symplastic connections to the entire seed coat. Active transport is required for EM to exit from living cells, as membranes present highly negative potential on the inside. The HMA-type transporter is the prime candidate for export of EM from the plant into the endosperm cavity. Transporters of the MTP family may also be associated with the regulation of the amount of EM entering the developing seed [192,193,194].

*The S content of the seed*—Yield and seed quality are related to the S content of the seed. S presents a great impact on the improvement of seed yield and quality. The S is transported from leaves, as the major source, during the various developmental phases of seed. Remobilization of S takes places in mature leaves and/or stems which are S transient storage pools. The information on the source-to-sink relationship during the time of seed development is hectic. The same holds true for the role of sulfate exported from the seed vacuoles during the seed developmental stage in order to maintain cellular homeostasis. SULTR3 and SULTR4 family transporters seem to be involved in S transportation mechanisms during seed development [195].

As regards the form of S delivered to seeds through the phloem, this is diverse; S is delivered as sulfate to pods of legumes, as GSH to rice grains, and as S-methylmethionine in wheat. Rice seeds receive an amino form of S as a nutritional form. Transportation of sulfate through sulfate transporters localized in the phloem contributes to the import of S in seeds. The S-reducing enzymes are also present in the developing seeds, resulting in the accumulation of reduced sulfur in mature seeds [195,196,197,198,199,200,201].

## 9. The Contribution of S to EM Agronomic Biofortification

The agronomic phytofortification activity to increase the EM content is associated with an increase in yield and quality. The sulfate salts of Fe, Cu, Zn, and Mn have been used for alleviating low contents of EM in seeds and edible parts, in comparison to EM complexed with synthetic ligands. The presowing application of S in the form of granular kieserite, in S-deficient soil, along with top dressing with magnesium sulphate heptahydrate and ammonium nitrate, was sufficient to achieve optimal grain yield with beneficial Fe, Mn, Zn, and Cu content in grain dry mass [202,203].

Fertilizer type along with application method influence the effectiveness of application on crop performance. The fertilizer formulation largely determines the EM phytoavailability. Toward increasing yield and nutritional quality of a crop, the fertilization of the rhizosphere with EM has been suggested as a sustainable strategy. However, foliar fertilization with EM often stimulates more nutrient uptake and efficient allocation in the edible plant parts compared with soil fertilization. Their combination is often the most effective method [9].

Foliar fertilization with FeSO_4_ is applied when low-Fe content is found in the soil. This treatment improved grain Fe concentration in wheat by about 28% in China, and 21% in Iran, whereas in Canada, it remained ineffective. By increasing soil N application, shoot and grain Fe contents significantly enhanced both under field and greenhouse conditions [204,205,206,207,208,209,210,211,212,213,214]. Application of Fe fertilizers alone either in inorganic (FeSO_4_) or in chelated form (e.g., Fe-EDDHA, Fe- EDTA or Fe-citrate) presented a small positive impact on increasing Fe content in grain, while in combination with soil N application, it enhanced by 47%. At a given Fe treatment, soil N supply can enhance shoot Fe concentrations by up to 70%. Foliar application of urea also improved grain Fe concentration [204,205,206,207,208,209,210,211,212,213,214].

Zn application as ZnSO_4_ increased grain yield and Zn content [202,215]. When plants are enriched with Zn, N should be taken care of, especially during the grain pouring phase, as N plays an important role in Zn uptake [216,217,218,219]. Zn and the other EMs concentrations increased after the use of appropriately high doses of N coupled with foliar fertilization with EMs [208,218,220,221].

Foliar applications of MnSO_4_.H_2_O and CuSO_4_.5H_2_O, three times and separately at three critical stages of wheat grain development, i.e., middle boot, early milk, and dough stages, along with increased dose of N as top-dressing prior to heading, enhanced nutrient uptake of N, Cu, and Mn, improved yield, and enriched mineral content of the wheat grains [9].

Another phytofortification example is based on the use of elemental S. Fertilizer granules can be enriched with 2% elemental sulfur (FES). A durum wheat crop that received the enriched fertilizer accumulated a higher amount of Fe compared to a conventional one. The fertilization with FES at sowing mobilized iron, thus providing more iron to the crop, and fortified the S status of the crop, too. The initiation of the fast stem elongation stage constituted a turning point. Prior to its initiation, the use of FES increased the iron concentration in the main stems, followed by an increase in the organic S concentration. Thereafter, the FES-crop presented plants with higher main stems and fewer tillers. At harvesting, all plant parts of the FES-crop were heavier, containing more iron and organic sulfur, and the obtained commercial yield of the FES-crop was higher by 27.3% [222].

*The need for integrated soil fertility management*—It is highlighted that the application of sulfate salts of Fe, Cu, Zn, and Mn is effective, or more effective, under an integrated soil fertility management approach. The form of the nutrients and the interactions between them can have positive, neutral, or negative effects on yields and nutrient use efficiencies. Various factors determine the success of agronomic phytofortification, depending on EM phytoavailability at different stages, along with efficient uptake and handling of EM by the plant. Soils with (multiple) EM deficiencies are nonresponsive to NPK, despite the addition of NPK fertilizers. Management of rhizosphere N and P is important for increasing the effectiveness of the applied EM. Wheat fertilization with NP fertilizer enriched in Zn has been effective in increasing the yield of wheat grain. The adequate N and P status of plants presents positive effects on root development, EM transport to the shoot, and remobilization of EM from leaves to seeds. In wheat crops, proper N application increased Zn and Fe contents in the grain endosperm. In sorghum and finger millet crops fertilized with blends of mineral NPK enriched with Zn, B and S, the N, P, Zn, B, and S contents were increased significantly, along with crop productivity [208,223,224].

Depending on the amount of the phytoavailable Zn, the rhizospheric P can stimulate root growth and Zn uptake, or the same application of P fertilizer can trigger Zn deficiency by precipitating the already hectic concentrations of Zn, as Zn phosphate is insoluble. Addition of P appears to induce Zn deficiency through dilution effects and interference with Zn translocation from the roots.

Symbioses with arbuscular mycorrhizal fungi (AMF) increases the uptake of nutrients, such as P, Fe, and Zn, that are sparingly soluble in rhizosphere. Mycorrhizal symbiosis modifies plant demands for reduced S and regulates the uptake, distribution, and assimilation of the sulfate accordingly [225]. Sulfate possibly regulates the expression of ZmNAS1, ZmNAS3, and ZmYS1 genes, revealing its potential role as signal molecule for the Fe homeostasis in AMF plants. The symbiosis with AMF prevented Fe-deprivation responses in the S-deprived maize plants. It seems that Fe was provided directly to the mycorrhizal plants through the fungal network [226]. Moreover, it has been proposed that the gene expression of the DMA exporter ZmTOM1 can be used as an early indicator for the establishment of a mycorrhizal relationship in maize [227]. The Fe uptake pathway seems to be regulated by sulfate supply in S-deprived maize plants. Moreover, a strong correlation seems to exist between the transcriptional regulation of the Fe-uptake pathway genes and the sulfate phytoavailability, and this holds true independently of the existence of mycorrhizal association or not. Sulfate is probably a key component of the signal transduction pathway that regulates the expression of the Fe-uptake pathway genes in maize plants [228].

## 10. Conclusions

The functional interactions of S with EM can be categorized into four groups: (1) effective EM bonding, (2) effective EM chelation, (3) effective EM phytoavailability, and (4) effective EM transport. Each category contributes to retaining EM metalome homeostasis, and then to the effective re-translocation of S and EM from source leaves to sink seeds. These interactions are based first on the properties of the sulfhydryl group, which contribute to the sequestration of the proper EM by a variety of molecules, enzymes, or chelators. The fact that NA is produced from Met and that NA serves as a ligand with carboxylic groups broadens the interaction spectrum with more capacities, along with chelation and transportation components. The allocation and the action of the discussed components explain why the sulfate salts of Fe, Zn, Cu, and Mn are efficient phytofortification agents. Coupled with ES and AMF, under integrated and balanced fertility management of the rhizosphere, it is anticipated that yield and its quality can be obtained. The existing knowledge on the interactions discussed above provides opportunities both for future improvements of the biofortification programs and actions, as well as for deeper understanding of the acting mechanisms about re-translocation from the source leaf to the specific sink. In a biofortification program, selected metabolites could be included such as S-containing amino acids or metabolites, alone or in combination with EM sulfate salts, toward sustaining plants’ resistance to transient deficiencies or stresses. This suggests further research on the physiological background of how the applied metabolites are translocated, handled, and re-translocated to the sink organ, and what the contribution of S is in the acting mechanisms.

## Data Availability

Not applicable.
